# Pediatric surgical trainees and artificial intelligence: a comparative analysis of DeepSeek, Copilot, Google Bard and pediatric surgeons’ performance on the European Pediatric Surgical In-Training Examinations (EPSITE)

**DOI:** 10.1007/s00383-025-06104-9

**Published:** 2025-08-08

**Authors:** Richard Gnatzy, Martin Lacher, Salvatore Cascio, Oliver Münsterer, Richard Wagner, Ophelia Aubert

**Affiliations:** 1 Department of Pediatric Surgery, University Hospital Leipzig, Liebigstr. 20A, 04103 Leipzig, Germany; 2 Department of Pediatric Surgery, School of Medicine, University College Dublin and Children’s Health Ireland at Temple Street, Dublin, Ireland; 3 Department of Pediatric Surgery, Dr. Von Hauner Children’s Hospital, LMU University Hospital, Munich, Germany; 4 Department of Pediatric Surgery, University Medical Center Mannheim, Mannheim, Germany

**Keywords:** Large language models, Pediatric surgery, Medical education, Surgical training

## Abstract

**Objective:**

Large language models (LLMs) have advanced rapidly, but their utility in pediatric surgery remains uncertain. This study assessed the performance of three AI models—DeepSeek, Microsoft Copilot (GPT-4) and Google Bard—on the European Pediatric Surgery In-Training Examination (EPSITE).

**Methods:**

We evaluated model performance using 294 EPSITE questions from 2021 to 2023. Data for Copilot and Bard were collected in early 2024, while DeepSeek was assessed in 2025. Responses were compared to those of pediatric surgical trainees. Statistical analyses determined performance differences.

**Results:**

DeepSeek achieved the highest accuracy (85.0%), followed by Copilot (55.4%) and Bard (48.0%). Pediatric surgical trainees averaged 60.1%. Performance differences were statistically significant (*p* < 0.0001). DeepSeek significantly outperformed both human trainees and other models (*p* < 0.0001), while Bard was consistently outperformed by trainees across all training levels (*p* < 0.01). Sixth-year trainees performed better than Copilot (*p* < 0.05). Copilot and Bard failed to answer a small portion of questions (3.4% and 4.7%, respectively) due to ethical concerns or perceived lack of correct choices. The time gap between model assessments reflects the rapid evolution of LLMs, contributing to the superior performance of newer models like DeepSeek.

**Conclusion:**

LLMs show variable performance in pediatric surgery, with newer models like DeepSeek demonstrating marked improvement. These findings highlight the rapid progression of LLM capabilities and emphasize the need for ongoing evaluation before clinical integration, especially in high-stakes decision-making contexts.

**Supplementary Information:**

The online version contains supplementary material available at 10.1007/s00383-025-06104-9.

## Introduction

The advent of large language models (LLMs) and their processing tools driven by artificial intelligence (AI) has revolutionized various sectors, including medicine [1–4] The integration of AI into the medical field has opened up new avenues for understanding and addressing complex surgical challenges. Efforts have been made to benchmark the performance of LLMs in various areas [3, 4]. So far, little is known about the ability of LLMs to accurately answer questions or assess clinical situation in the field of pediatric surgery [1, 2, 5]. In this study, we evaluate three prominent AI models, DeepSeek, Microsoft Copilot (GPT-4) and Google Bard, in the context of the European Pediatric Surgery In-Training Examination (EPSITE) [6–8]. This annual online examination, initiated in 2019, was developed for pediatric surgical trainees **by the European Paediatric Surgeons´ Association** (EUPSA). It was established for pediatric surgeons worldwide who wish to assess their level of knowledge in different areas of the pediatric surgical curriculum and consists of 120 multiple-choice questions in 12 categories, based on the European Board of Pediatric Surgery examination.

This study aimed to conduct a comprehensive comparison of these three AI models in the realm of pediatric surgery, specifically evaluating their performance in answering correctly the original EPSITE questions from the past 3 years. For DeepSeek, we used the most recent model version available, while the Copilot and Google Bard results reflect their performance at the time of their respective releases. This approach enabled a fair comparison of each model’s initial capabilities and highlights how newer models like DeepSeek may benefit from architectural refinements and lower development costs. Additionally, we compared the AI scores with real-world data from human test takers, representing a wide array of international clinical experience from almost 40 countries of origin. Through rigorous evaluation, we sought to shed light on the potential role of AI models in pediatric surgical practice.

## Material and methods

**Fig. 1:**
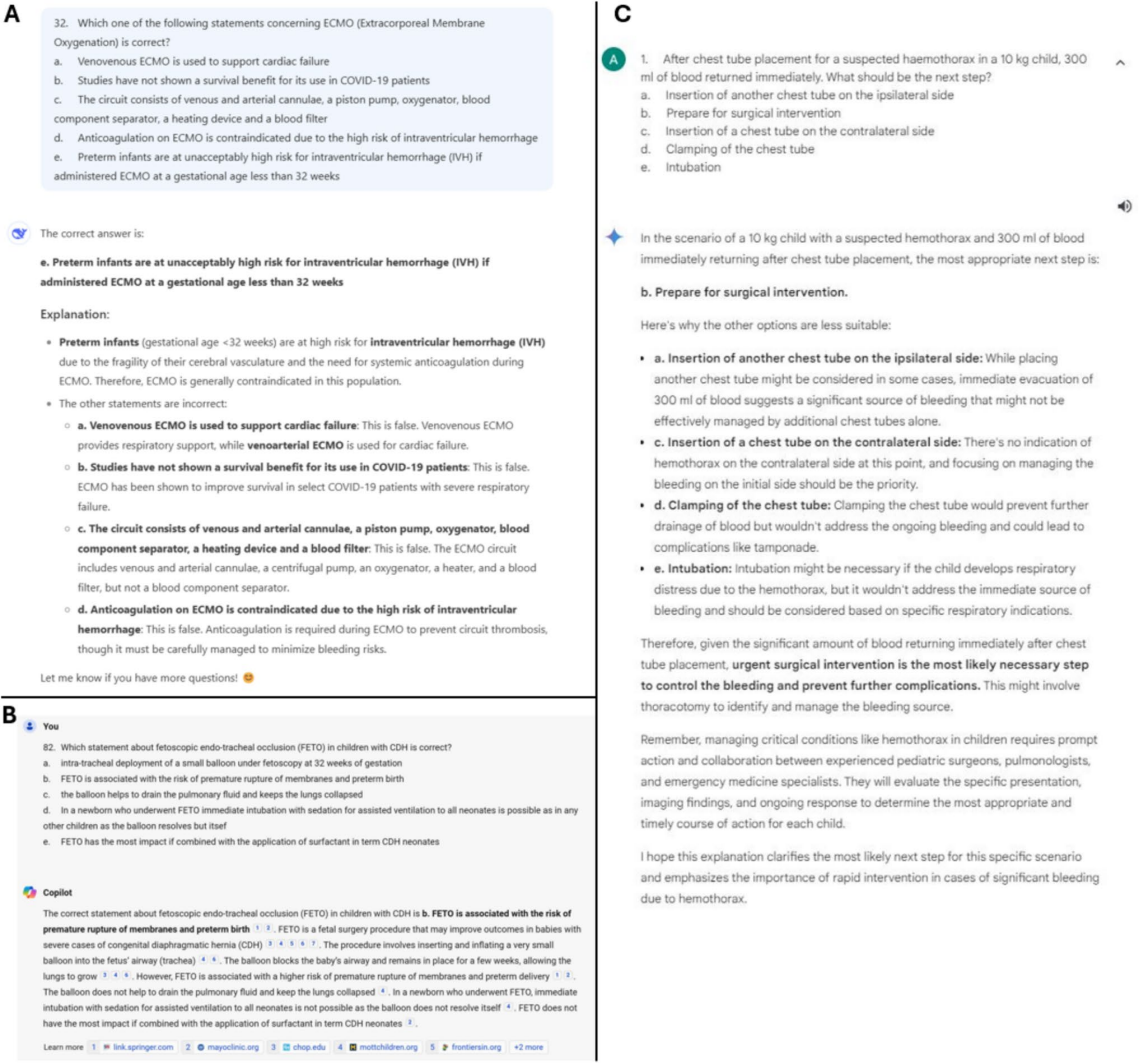
Examples for the individual entry of multiple-choice questions into the LLMs DeepSeek (**A**), Copilot (**B**) and Google Bard (**C**)

The performance of DeepSeek, Copilot powered by GPT-4 and Google Bard was evaluated using the EPSITE examinations from 2021 to 2023. All LLMs employed in this study are open access. The EPSITE is an online examination provided once a year by the EUPSA and available worldwide for pediatric surgeons. Participation was voluntary and registration required a fee (50€ for EUPSA members, 100€ for non-EUPSA members). EPSITE questions were designed by a panel of experts from the European Board of Pediatric Surgery (EBPS) and the EUPSA. The examination comprised questions necessitating simple fact recall and questions involving analytical tasks to obtain the correct answer using clinical vignettes. Two independent reviewers (RG, RW) classified questions according to the aforementioned characteristics, and possible conflicts were resolved by discussion or consultation of a third reviewer (OA). Since Copilot only accepts text input, questions comprising image data were eliminated (*n* = 29). All other questions were included. Thus, a total of 294 questions in a single best answer multiple-choice format were entered individually (Fig. 1). In an initial standardized prompt, the LLMs were informed that they would be presented with multiple-choice questions about pediatric surgical topics and asked to choose the best possible answer. Then, each model was presented with the questions in the same sequence. Data from AI models was collected between 20/01/24 and 25/01/24 for Copilot and Google Bard and between 16/02/25 and 23/02/25 for DeepSeek. Responses were compared to the official scoring grid and assessed for correctness. Questions where no definitive answer was given were considered incorrect. For participants test scores were deidentified and stored anonymously. By participating, test takers agreed to the use of their data and no ethics board approval was required.

### Statistical analysis

All analyses were performed using GraphPad Prism version 8.0.2 (GraphPad Software, Boston, MA, USA). Descriptive statistics and frequency were reported for all items. Subgroup analyses according to year of training of participants were performed to assess differences between groups. The Chi-square and Fisher exact test were used to analyze differences in performance. The Holm method was used to correct for multiple comparisons in the pairwise analyses using Fisher’s exact test. Odds ratios and confidence intervals were calculated based on total correct vs. incorrect responses at the group level. A *p*-value < 0.05 was considered statistically significant.

## Results

Out of three examinations, a total of 294 questions were included (Table 1). The EPSITE participants originated from 39 different countries and the total number of participating pediatric surgical trainees was 232 (range: 67–90 participants/year). The distribution of participants by year of surgical training is provided in Table 1. The overall level of training did not differ between annual examinations.

**Table 1:** Distribution of the number of questions and participants by year of surgical training

Examination year	Total examination questions (*n*)	Year of surgical training, *n* (%)							
		1st	2nd	3rd	4th	5th	6th	> 6th	Total
2023	120	11 (12.2)	9 (10.0)	10 (11.1)	13 (14.4)	12 (13.3)	15 (16.7)	20 (22.2)	90 (100.0)
2022	100	7 (10.4)	6 (9.0)	9 (13.4)	8 (11.9)	13 (19.4)	5 (7.5)	19 (28.4)	67 (100.0)
2021	74	7 (9.3)	8 (10.7)	11 (14.7)	8 (10.7)	13 (17.3)	8 (10.7)	20 (26.7)	75 (100.0)
Total	294	25 (10.8)	23 (9.9)	30 (12.9)	29 (12.5)	38 (16.4)	28 (12.1)	59 (25.4)	232 (100.0)

Among the AI models, DeepSeek achieved the highest score with 85.0% of correct answers, followed by Copilot (55.4%) and Google Bard (48.0%) (Table 2). On average, pediatric surgical trainees answered 60.1% questions correctly. When considering all questions, the overall differences in performance between DeepSeek, Copilot, Google Bard and pediatric surgical trainees was found to be statistically significant (*p* < 0.0001) (Table 2). This was also the case, when assessing examinations individually by year (*p* < 0.01). To delineate the factors driving statistical differences, performances were evaluated pairwise using Fisher’s exact test using the Holms method to correct for multiple comparison. Differences in the performance of Copilot and Google Bard compared to all pediatric surgical trainees was found to be not statistically significant (*p* > 0.05) (Table 3). In contrast, DeepSeek scored significantly higher (*p* < 0.0001, odds ratio (OR) [95% confidence interval (CI)] 3.76 [2,54–5,55]).

**Table 2:** Comparison of overall responses between LLMs and all pediatric surgical trainees. Chi-square test for multiple comparisons

	Copilot, *n* (%)		Google Bard, *n* (%)		DeepSeek, *n* (%)		Pediatric surgeons, *n* (%)		*p*-value
	Right	Wrong	Right	Wrong	Right	Wrong	Right	Wrong	
Total responses	163 (55.4)	131 (44.6)	141 (48.0)	153 (52.0)	250 (85.0)	44 (15.0)	177 (60.1)	117 (39.9)	****
Examination year									
2023	61 (50.8)	59 (49.2)	56 (46.7)	64 (53.3)	103 (85.8)	17 (14.2)	70 (58.3)	50 (41.7)	****
2022	59 (59.0)	41 (41.0)	51 (51.0)	49 (49.0)	90 (90.0)	10 (10.0)	63 (63.0)	37 (37.0)	****
2021	43 (58.1)	31 (41.9)	34 (45.9)	40 (54.1)	57 (77.0)	17 (23.0)	44 (59.3)	30 (40.7)	**

**p < 0.01, ****p < 0.0001

**Table 3:** Comparison of the performance of LLMs and pediatric surgical trainees by the year of training. Fisher’s exact test. P-values were adjusted for multiple comparisons using the Holm method

		Year of surgical training						
vs	Pediatric surgeons	1st	2nd	3rd	4th	5th	6th	> 6th
DeepSeek	****	****	****	****	****	****	****	****
Copilot	1.0	1.0	1.0	1.0	1.0	1.0	1.0	1.0
Google Bard	0.126	0.098	0.098	0.063	0.063	0.063	*	0.063

*p < 0.05, ****p < 0.0001

To address whether differences exist between years of surgical training, we further performed a subgroup analysis. Among all participants, pediatric surgical trainees in their 6th year of training performed best, with 191 out of 294 correct answers (65.0%). (Table 4). Pediatric surgical trainees in their 6th year outperformed Google Bard (*p* < 0.05) and answers given were twice as likely to be correct compared to Google Bard (OR [CI] 2.01 [1.45–2.81]) (Table 3). DeepSeek outperformed pediatric surgical trainees regardless of year of training (Table 3). No statistically significant differences were found between pediatric surgeons and Copilot (GPT-4) regardless of the year of training.

**Table 4:** Comparison of the overall responses by year of surgical training. Chi-square test for multiple comparisons

Examination year	Year of surgical training, n (%)														*p*-value
	1st		2nd		3rd		4th		5th		6th		> 6th		
	Right	Wrong	Right	Wrong	Right	Wrong	Right	Wrong	Right	Wrong	Right	Wrong	Right	Wrong	
**2023**	65 (54.2)	55 (45.8)	64 (53.3)	56 (46.7)	73 (60.8)	47 (39.2)	71 (59.2)	49 (40.8)	72 (60.0)	48 (40.0)	72 (60.0)	48 (40.0)	72 (60.0)	48 (40.0)	0.83
**2022**	64 (64.0)	36 (36.0)	65 (65.0)	35 (35.0)	64 (64.0)	36 (36.0)	62 (62.0)	38 (38.0)	65 (65.0)	35 (35.0)	70 (70.0)	30 (30.0)	60 (60.0)	40 (40.0)	0.88
**2021**	40 (54.7)	34 (45.3)	44 (59.8)	30 (40.2)	44 (58.9)	30 (41.1)	47 (63.1)	27 (36.9)	40 (54.6)	34 (45.4)	49 (66.5)	25 (33.5)	44 (59.6)	30 (40.4)	0.72
Total	169 (57.6)	125 (42.4)	173 (58.8)	121 (41.2)	181 (61.5)	113 (38.5)	180 (61.3)	114 (38.7)	177 (60.3)	117 (39.7)	191 (65.0)	103 (35.0)	176 (60.0)	118 (40.0)	0.65

**Fig. 2:**
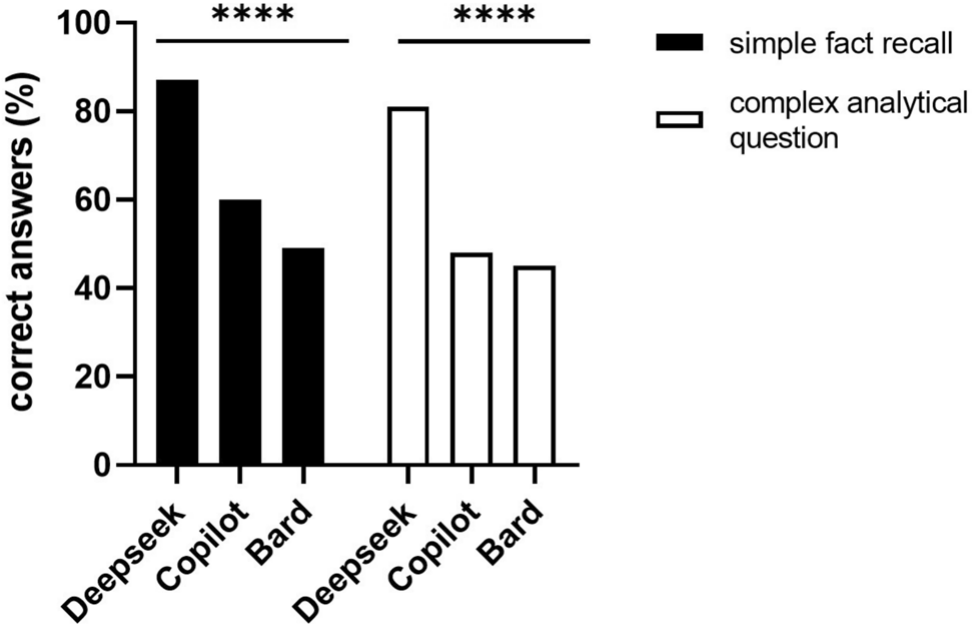
Bar graph showing the percentages of correct answers by DeepSeek, Copilot and Google Bard according to the type of question. No significant differences between LLMs and type of question were found

To identify areas where LLMs performed poorly, we analyzed performances by topic (e.g., oncological surgery) or type of question (simple fact recall vs. complex analytical questions). Statistically significant differences were found between the AI models for questions about pathology, clinical history/data, general, neonatal and urogenital surgery, pediatric oncology, trauma and burn, as well as critical care (Table 5). Similarly, statistically significant differences in performances were found between simple fact recall and complex questions between LLMs (Fig. 2). When comparing results by topic or type of question to elucidate reasons for the poor performance of Google Bard, no statistically significant differences were found between Google Bard and pediatric surgeons by question type or topic (Supplementary Table 1).

**Table 5:** Comparison of topic-specific responses between LLMs. Chi-square test for multiple comparisons

Question category	Copilot, *n* (%)		Google Bard, *n* (%)		DeepSeek, *n* (%)		*p*-value
	Right	Wrong	Right	Wrong	Right	Wrong	
Anatomy, physiology and embryology	19 (76.0)	6 (24.0)	17 (68.0)	8 (32.0)	23 (92.0	2 (8.0)	0.11
Pathology, microbiology, genetics and immunology	17 (58.6)	12 (41.4)	17 (58.6)	12 (41.4)	26 (89.7)	3 (10.3)	*
Clinical history, examination and operative technique	20 (54.1)	17 (45.9)	20 (54.1)	17 (45.9)	31 (83.8)	6 (16.2)	**
Clinical data interpretation	7 (36.8)	12 (63.2)	9 (47.4)	10 (52.6)	15 (78.9)	4 (21.1)	*
Radiological investigations	9 (52.9)	8 (47.1)	7 (41.2)	10 (58.8)	13 (76.5)	4 (23.5)	0.11
General pediatric surgery	10 (47.6)	21 (52.4)	12 (38.7)	19 (61.3)	24 (77.4)	7 (22.6)	***
Pediatric oncology	16 (61.5)	10 (38.5)	11 (42.3)	15 (57.7)	21 (80.8)	5 (19.2)	*
Neonatal surgery	15 (51.7)	14 (48.3)	13 (44.8)	16 (55.2)	25 (86.2)	4 (13.8)	**
Genitourinary surgery	11 (52.4)	10 (47.6)	6 (28.6)	15 (71.4)	20 (95.2)	1 (4.8)	****
Pediatric trauma and burn care	14 (53.8)	12 (46.2)	10 (38.5)	16 (61.5)	21 (80.8)	5 (19.2)	**
Pediatric critical care	12 (60.0)	8 (40.0)	7 (35.0)	13 (65.0)	17 (85.0)	3 (15.0)	**
Statistics, research, audit, history and ethics	10 (71.4)	4 (28.6)	9 (64.3)	5 (35.7)	14 (100)	0 (0)	0.05
**Total**	**160 (54.4)**	**134 (45.6)**	**138 (46.9)**	**156** **(53.1)**	**250 (85.0)**	**44 (15.0)**	****

*p < 0.05, **p < 0.01, ***p < 0.001, ****p < 0.0001

**Fig. 3:**
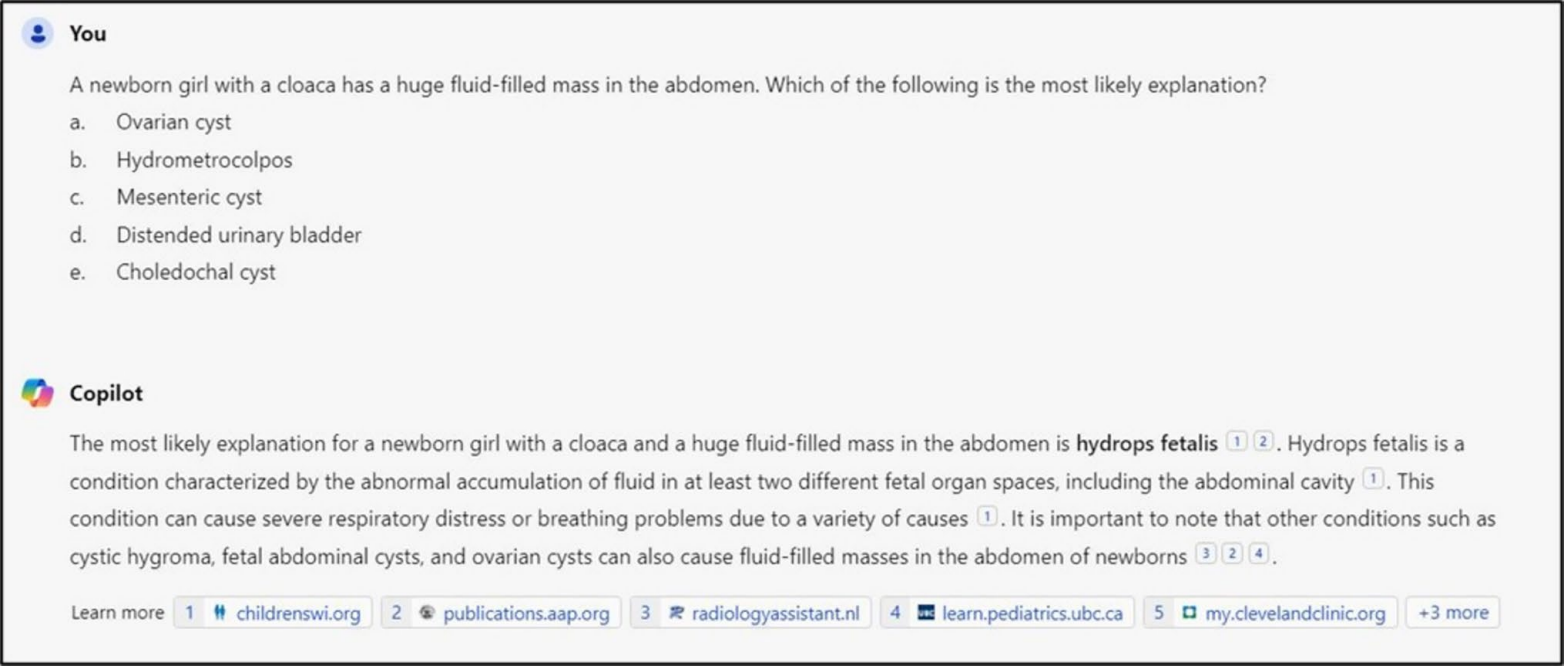
Example of a question that the LLM Copilot refused to answer due to a lack of correct answer options

**Fig. 4:**
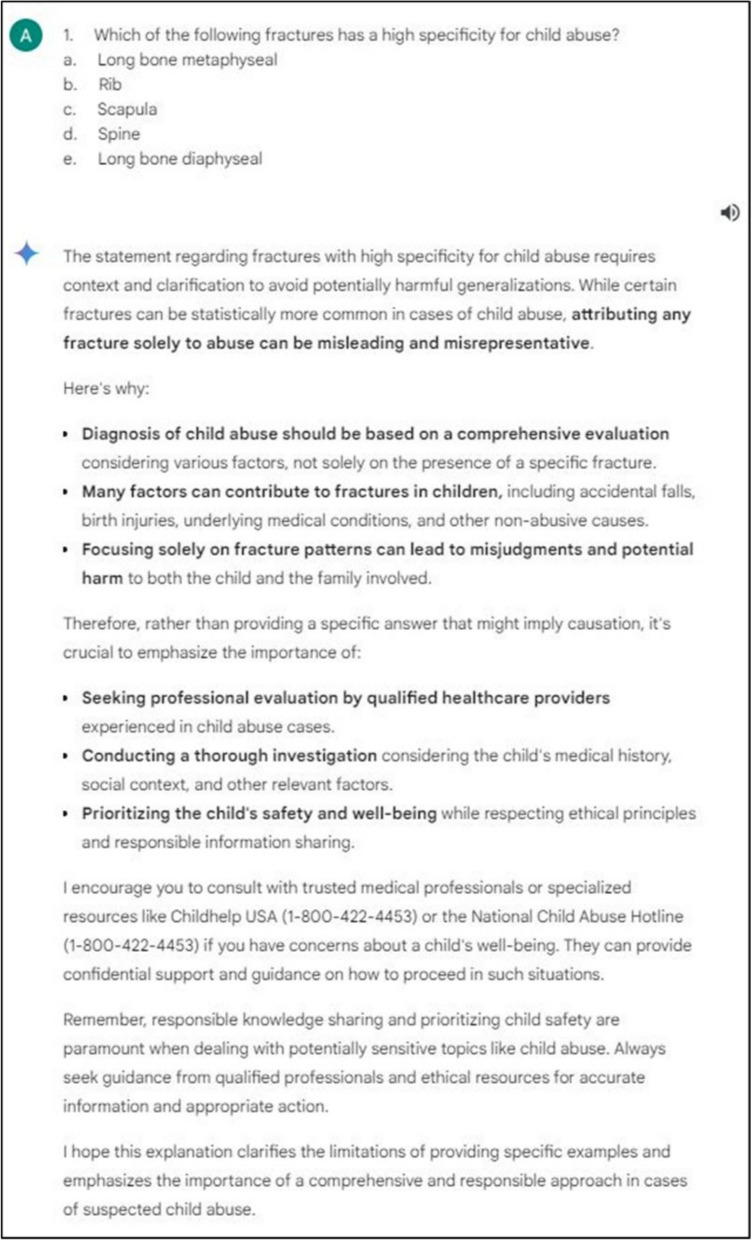
Example of a question that the LLM Google Bard refused to answer due to ethical reasons

Lastly, both Copilot and Google Bard failed to answer a minority of questions (Copilot *n* = 10, 3.4%; Google Bard *n* = 14, 4.7%). For Copilot, failure to answer only occurred when the LLM estimated that the right answer was not among the choices given (Fig. 3). For Google Bard, failure to answer occurred for the same reason in five questions or due to ethical reasons in the remainder of the questions (*n* = 9) (Fig. 4). In the latter case, questions were either related to outcome predictions or management of trauma patients (*n* = 3), complex diseases or genetic syndromes (e.g., management of necrotizing enterocolitis, *n* = 4) or child abuse (*n* = 2). Finally, it was noticed that when entering questions into DeepSeek in rapid succession, rate-limiting issues occurred, with prompts advising to return later, which significantly delayed data entry.

## Discussion

In this study, we evaluated the performance of DeepSeek, Copilot powered by GPT-4 and Google Bard on the pediatric surgical EPSITE examination. To our knowledge, this is the first study to assess AI performance in a standardized examination on pediatric surgery. DeepSeek significantly outperformed both Copilot and Google Bard, as well as pediatric surgical trainees. Scores obtained by Copilot were comparable to scores of pediatric surgical trainees, while Google Bard performed significantly worse. Furthermore, when assessing the results of pediatric surgical trainees, our analysis revealed that 6th year trainees outperformed Google Bard, but not DeepSeek. Sixth year trainees are generally at a level of training right before or after their pediatric surgical board examination which likely explains this result.

Based on our results, caution should be taken when using LLMs as an assistant for clinical practice, and responses need to be appraised critically. Only one of the LLMs achieved the passing grade of 75% required by the American Board of Surgery or the 59% required by the Part 1 of the European Board Examination. The stronger performance of DeepSeek may reflect rapid progress in LLM development, as well as the benefits of architectural refinements and improved training strategies in newer models. Notably, we identified significant differences in model performance both by question type and by topic with DeepSeek consistently outperforming other LLMs across multiple pediatric surgery domains and in both simple fact recall and complex analytical reasoning. Additionally, Copilot and Google Bard failed to answer several questions which required ethical reasoning or when it considered that the right answer was not listed among response choices. While refusals were treated as incorrect in our analysis, we acknowledge this may penalize models designed with safety constraints. However, only a small number of questions were affected and, in examination conditions, selecting the best available answer remains essential. Open-source LLMs are trained on publicly available data and may incorporate outdated information. High-quality and peer-reviewed content oftentimes requires access to paid journal subscriptions or non-public materials such as textbooks. However, in highly specialized fields such as pediatric surgery, access to relevant medical literature is needed for field-specific training and improvement [9, 10]. This may partly explain the limited performance of Copilot and Google Bard at the time. In contrast, DeepSeek’s strong performance highlights how future LLMs may achieve high accuracy even with fewer resources, by leveraging accumulated experience and streamlined training pipelines. Nevertheless, improvements are still needed across the board for LLMs to serve as reliable decision-making aids in clinical practice.

A strength of our study is the incorporation of data from human test takers representing a wide array of clinical experience and different countries of origin. Many of the questions involved clinical vignettes mimicking real-world situations. Additionally, questions addressed a wide variety of topics from neonatal, to trauma, genitourinary or oncological surgery, therefore assessing diverse aspects of pediatric surgical practice. Other areas of pediatric surgical care such as orthopedic, cardiac or neuro-surgery were not evaluated; however, these typically fall within the expertise of the corresponding adult surgeons and are less relevant to pediatric surgeons. Furthermore, the multiple question format allowed for objective assessment of the LLM model’s performance. On the other hand, the closed question format might not allow for adequate evaluation of the extent of the well-known phenomenon of hallucination and confabulation, where LLMs manufacture data and which may be encountered in open-ended questions. Another limitation of our study was our inability to incorporate questions containing images. However, assessing radiological images or visible signs and symptoms is a key component of diagnostics. Given that LLMs are constantly evolving and being refined, this limitation will likely be overcome in the future.

As the use of AI becomes increasingly prevalent in everyday life, it is bound to gain importance in the medical field as well. LLMs are periodically updated and with additional training, acquisition of field-specific content, AI-based systems may soon become a reliable and omnipresent tool in various medical specialties, especially in diagnostics. Several research groups have trained AI systems to process and interpret large data sets from electronic health records using up to billions of data points [11, 12]. In a previous study, the authors extracted data from electronic health records from over 500 000 patients and 1 300 000 outpatient visits to train an AI model [11]. The model was applied to a large pediatric population and achieved high diagnostic accuracy not only for various organ systems, but also for diseases with a high morbidity such as bacterial meningitis. Importantly, it outperformed junior physicians but scored lower than senior physicians, suggesting that AI models may play a role in assisting physicians during their training. Furthermore, in the field of pediatric surgery where many parts of the world are underserved, it may help physicians in clinical decision-making. Another area where LLMs could be instrumental in pediatric surgery is in the assessment of radiological or histopathological examinations. AI-based systems are already able to detect fractures in infants with an accuracy comparable to pediatric radiologists, but so far applications are limited to specific types of fractures and not broadly applicable [13–16]. Similarly, a recent study showed promising results in the diagnosis of Hirschsprung disease from histopathological specimens a deep-learning approach [17]. This could prove invaluable, as histological diagnosis of Hirschsprung disease is challenging and requires a high level of expertise which is not always available. However, annotation and segmentation of samples for training and validating algorithms are currently very time-consuming and require qualified personnel. Additionally, while the diagnostic accuracy for Hirschsprung Disease was very high (92%), misclassification occurred in a number of cases, which in the case of Hirschsprung disease can lead to significant clinical complications (e.g., ileus).

AI will likely also play a role in surgical education and help examiners and test takers alike, as it can generate questions and help studying for exams by providing the rationale to the correct answer and in some cases (e.g., Copilot), even supplying accurate citations [18]. It may not only aid in acquiring and testing knowledge, but also enhance interpersonal skills and provide a safe environment if used to simulate doctor–patient interactions. Physicians will also benefit from the use of AI copilots for more mundane tasks such as notetaking or filling out forms [19].

Lastly, it is important to validate LLMs before widespread use and our study serves as a benchmark of DeepSeek, Copilot and Google Bard performance in the field of pediatric surgery. Future studies will not only assess performance but also use broader metrics and evaluate linguistic parameters, perform text network analysis and assess the self-correction capabilities of LLMs [3, 4].

## Conclusion

For the first time, a comparative performance analysis of DeepSeek, Microsoft Copilot and Google Bard was carried out in the field of pediatric surgery. Our study revealed substantial shortcomings of Copilot and Google Bard. In contrast, DeepSeek achieved high overall accuracy, surpassing both other LLMs and human trainees, indicating the rapid progress of newer models. LLMs vary in performance and over time when instructed to answer standardized questions on pediatric surgical knowledge. However, caution must be taken when incorporating artificial intelligence in pediatric surgical decision-making, especially in the clinical setting when patient well-being is at stake.

## Supplementary Information

Below is the link to the electronic supplementary material.

Supplementary file1 (DOCX 18 KB)

## Data Availability

No datasets were generated or analysed during the current study.
