# Supplementary material for: Pediatric surgical trainees and artificial intelligence: a comparative analysis of DeepSeek, Copilot, Google Bard and pediatric surgeons’ performance on the European Pediatric Surgical In-Training Examinations (EPSITE)
Source: Pediatr Surg Int. 2025 Aug 8;41(1):247. doi: 10.1007/s00383-025-06104-9 (PMC12334501; doi:10.1007/s00383-025-06104-9)
Supplement: Supplementary file 1 — Supplementary file1 (DOCX 18 KB) [file 383_2025_6104_MOESM1_ESM.docx]

**Supplementary Table 1:** Comparison of type and topic of questions between pediatric surgeons and DeepSeek. Fisher’s exact test. **p<0.5, **p<0.01, ***p<0.001, ****p<0.0001.*

| **Question category** | **Pediatric Surgeons**  **n (%)** | | **DeepSeek,  n (%)** | | **p-value** |
| --- | --- | --- | --- | --- | --- |
|  | right | wrong | right | wrong |  |
| Simple questions | 109  (59.9) | 73  (40.1) | 159  (87.4) | 23  (12.6) | **** |
| Complex analytical questions | 65  (58.0) | 47  (42.0) | 91  (81.3) | 21  (18.7) | *** |
| Anatomy, Physiology and Embryology | 17  (68.0) | 8  (32.0) | 23 (92.0) | 2  (8.0) | 0.07 |
| Pathology, Microbiology, Genetics and Immunology | 17 (58.6) | 12  (41.4) | 26 (89.7) | 3 (10.3) | * |
| Clinical History, Examination and Operative Technique | 24  (64.9) | 13  (35.1) | 31 (83.8) | 6 (16.2) | 0.10 |
| Clinical Data Interpretation | 11  (57.9) | 8  (42.1) | 15 (78.9) | 4 (21.1) | 0.29 |
| Radiological Investigations | 10  (58.8) | 7  (41.2) | 13 (76.5) | 4 (23.5) | 0.46 |
| General Pediatric Surgery | 15  (48.4) | 16  (51.6) | 24 (77.4) | 7 (22.6) | * |
| Pediatric Oncology | 15  (57.7) | 11 (42.3) | 21 (80.8) | 5 (19.2) | 0.13 |
| Neonatal Surgery | 17  (58.6) | 12  (41.4) | 25 (86.2) | 4 (13.8) | * |
| Genito Urinary Surgery | 12  (57.1) | 9  (42.9) | 20 (95.2) | 1  (4.8) | ** |
| Pediatric Trauma and Burn Care | 15  (57.7) | 11  (42.3) | 21 (80.8) | 5 (19.2) | 0.13 |
| Pediatric Critical Care | 12  (60.0) | 8  (40.0) | 17 (85.0) | 3 (15.0) | 0.15 |
| Statistics, Research, Audit, History and Ethics | 9  (64.3) | 5  (35.7) | 14 (100) | 0  (0) | * |
